# *iMS2Flux* – a high–throughput processing tool for stable isotope labeled mass spectrometric data used for metabolic flux analysis

**DOI:** 10.1186/1471-2105-13-295

**Published:** 2012-11-12

**Authors:** C Hart Poskar, Jan Huege, Christian Krach, Mathias Franke, Yair Shachar-Hill, Björn H Junker

**Affiliations:** 1 Department of Physiology and Cell Biology, Leibniz Institute of Plant Genetics and Crop Plant Research (IPK), 06466, Gatersleben, Germany; 2 Plant Biology Department, Michigan State University, East Lansing, MI, 48824, USA; 3 Institute of Pharmacy, Martin-Luther-University Halle-Wittenberg, 06120, Halle, Germany

## Abstract

**Background:**

Metabolic flux analysis has become an established method in systems biology and functional genomics. The most common approach for determining intracellular metabolic fluxes is to utilize mass spectrometry in combination with stable isotope labeling experiments. However, before the mass spectrometric data can be used it has to be corrected for biases caused by naturally occurring stable isotopes, by the analytical technique(s) employed, or by the biological sample itself. Finally the MS data and the labeling information it contains have to be assembled into a data format usable by flux analysis software (of which several dedicated packages exist). Currently the processing of mass spectrometric data is time-consuming and error-prone requiring peak by peak cut-and-paste analysis and manual curation. In order to facilitate high-throughput metabolic flux analysis, the automation of multiple steps in the analytical workflow is necessary.

**Results:**

Here we describe *iMS2Flux,* software developed to automate, standardize and connect the data flow between mass spectrometric measurements and flux analysis programs. This tool streamlines the transfer of data from extraction via correction tools to ^13^C-Flux software by processing MS data from stable isotope labeling experiments. It allows the correction of large and heterogeneous MS datasets for the presence of naturally occurring stable isotopes, initial biomass and several mass spectrometry effects. Before and after data correction, several checks can be performed to ensure accurate data. The corrected data may be returned in a variety of formats including those used by metabolic flux analysis software such as *13CFLUX*, *OpenFLUX* and *13CFLUX2*.

**Conclusion:**

*iMS2Flux* is a versatile, easy to use tool for the automated processing of mass spectrometric data containing isotope labeling information. It represents the core framework for a standardized workflow and data processing. Due to its flexibility it facilitates the inclusion of different experimental datasets and thus can contribute to the expansion of flux analysis applications.

## Background

In metabolic flux analysis (MFA), fluxes are defined as the flows of molecules between different metabolite pools catalyzed by the corresponding enzymes and/or transporters. MFA allows the determination of *in vivo* fluxes in a given metabolic network. To achieve this, MFA combines a stoichiometric model, as the mathematical representation of the metabolic network, and measurement data from isotope labeling experiments [1,2]. Taken together the model and the metabolite labeling information facilitate the calculation of *in vivo* fluxes not accessible by direct techniques. However, MFA is characterized by certain technical and conceptual challenges, for example the exact quantification of the stable isotopes introduced to the system under investigation [3-5]. The determination of the amount of label taken up is complicated by several factors: (i) naturally occurring stable isotopes (NOIs) of almost all elements found in metabolites, including ^13^C: 1.1%, ^2^H 0.0115%, ^17^O 0.038%, ^18^O 0.2% ^15^N 0.366%, and ^34^S: 4.2%, [6,7]; (ii) additional elements with stable isotopes introduced by derivatization such as ^29^Si or ^30^Si, natural abundance 4.7% and 3.1%, respectively [8-11]; (iii) proton gain or loss during mass spectrometric analysis. The extent of this depends on the chemical nature of the metabolites, the mass spectrometric technique employed, and the sample composition, e.g. the McLafferty rearrangement [12]; and (iv) dilution by the original biomass of the biological sample prior to the feeding of isotope labeled tracers [13,14]. To avoid systematic errors in the determined fluxes, the labeling levels detected have to be corrected for these biases.

**Figure 1:**
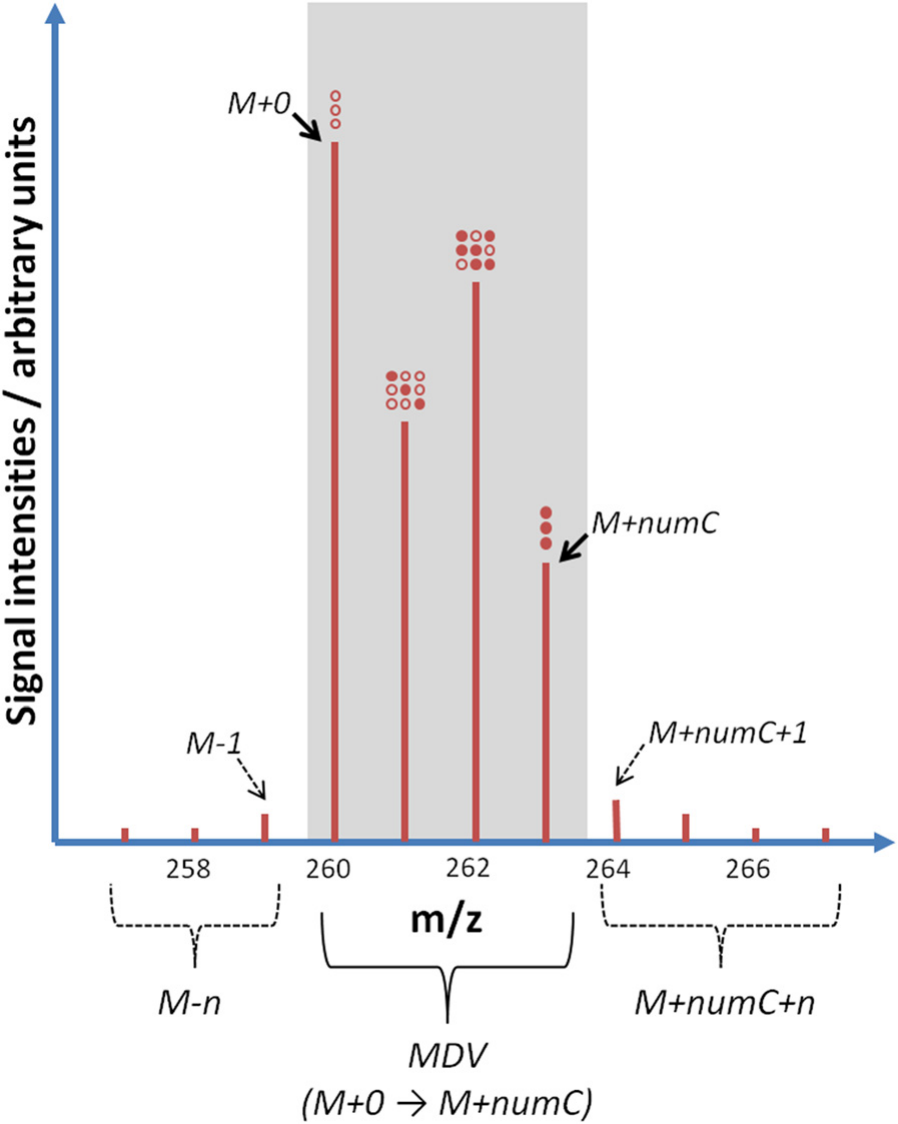
**Illustration of the mass isotopomer distribution vector (MDV) of a three carbon compound, e.g. alanine.** The signals with the grey background, the MDV, comprise the fractions of completely unlabeled (M+0), singly labeled (M+1), doubly labeled (M+2), and completely labeled (M+numC in general, i.e. M+3 in this example) analyte. The preceding (M-n) and following (M+numC+n) masses, which form a boundary around the analyte masses, are indicated.

In other “omic” technologies higher throughput rates have evolved through the development of more efficient workflows. This involves automating and integrating different steps of the analytical process. The example of metabolomics is instructive where sample preparation, spectrometric analysis and data processing are now routinely integrated [15-18]. As parallel processing of samples and automated instrumental analyses have become common, accurate processing of labeling data can limit the throughput of flux analysis or profiling. A typical isotope tracer experiment can result in multiple chromatograms, each containing mass spectrometric (MS) information on dozens of analytes, each of which can yield mass isotopomer patterns of multiple fragments. The entirety of mass isotopomers of a fragment ion is called the mass isotopomer distribution vector (MDV) [4], cf. Figure 1.

The capability for high–throughput labeling studies was recently demonstrated for cell cultures of mammalian cells or microbes. Steady state flux analysis (one time point) was performed with up to 135 strains of *Bacillus subtilis*[19] using one labeling substrate, as well as 92 strains of *Escherichia coli*[20] using four different labeling substrates and the isotope labeling data of ~12 amino acids were analyzed. Time course labeling experiments using multiple labeling substrates were performed with human cells, with two treatments, [21] or *Clostridium acetobutylicum*[22], at ~7 time points, with up to 20 analytes measured by liquid chromatography – mass spectrometry (LC-MS). However these examples of high-throughput experiments are still the exception. In general the throughput has not dramatically increased in the last several years, although the number of analytes for which labeling can accurately be detected in a single run is now potentially much larger [9,23-26] and the value of multiple biological replicates using different label combinations has been demonstrated [27-29].

A range of useful software is available that perform different aspects required for MFA including MS data extraction [30,31], data correction [11,32], model development and analysis [33,34], see Table 1. So far only one unified framework exists, *FiatFlux*[35], which attempts to combine all of the above mentioned aspects.

**Table 1:** **Comparison of *iMS2Flux *and other available MS correction tools**

**Tool**	***MSCorr***	***CORRECTOR***	***iMS2Flux***	***FiatFlux***	***corrMatGen (OpenFLUX)***
**MS Data Extraction**^**a**^	-	-	-	√	-
**MS Data Quality Check**	√	-	√	√	-
**MS Data Correction Methods:**					
**NA / NOI**	√	√	√	√	√^e^
**OBM**	-	-	√	√	-
**Proton loss/gain**	-	-	√/√	-	-
**Output ready for use in Flux-Software:**					
**13CFlux/ 13CFlux2**	-	-	√/√	-	-
**OpenFlux**	-	-	√	-	√
**FiatFlux**	-	-	-	√	-
**Model development & analysis**	-	-	-	√	√
**Quantification of isotope enrichment**	-	√	√	-	-
**High-throughput capability**^**b**^	-	√	√	√	-
**Multiple labeling substrate / isotope**^**c**^	√/-	√/√	√/√	-	√/√
**Analytical platform**	GC	GC/LC	GC/LC	GC	GC/LC
**additional software required**^**d**^	proprietary	-	free	proprietary	proprietary
**Full source code available**	√	√	√	-	-

^a^ directly from chromatogram files like net-cdf files.

^b^ multiple compounds in multiple chromatograms.

^c^ the data processing is independent from the utilized labeling substrate (e.g. uniformly labeled or different positional labeling) and can be adapted to other elements then carbon (e.g. nitrogen, oxygen).

^d^ requires additional software in order to be used like MATLAB or PERL.

^e^ functional support is provided but not directly integrated.

Abbreviatons: NA - Natural Abundance; NOI - Naturally Occuring stable Isotopes; OBM - Original Biomass; GC - Gas Chromatography; LC - Liquid Chromatography.

Consequently the automation of MS data processing, examination and correction of large and heterogeneous tracer experimental datasets would provide a more efficient workflow and bring MFA significantly closer to being a high–throughput technology. Here we describe *iMS2Flux*, a tool that provides a framework for a standardized workflow to automatically process MS data from isotope tracer experiments. It includes data quality checks as well as correcting the MS data for NOIs, proton loss or gain and original biomass. Finally the processed data can be delivered in formats used by MFA dedicated modeling software.

## Implementation

**Figure 2:**
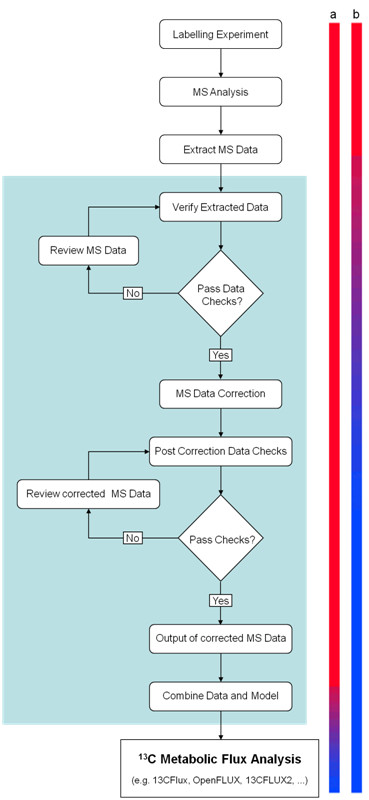
**Overview of the MFA workflow.** This scheme of the data processing steps highlight (blue background) those implemented in *iMS2Flux*. The bars on the right indicate the reduction of “hands on time” (in red) for the scientist by automation (in blue) of the MS data processing, bringing MFA one step closer towards high-throughput. Colour bar a) illustrating the standard workflow and colour bar b) illustrating the automated workflow with *iMS2Flux.*

*iMS2Flux* has been developed in PERL (the Practical Extraction and Reporting Language) which is available for all major computing platforms. *iMS2Flux* consists of five major parts: data input, data checking, data correction, post correction checks, and output (cf. Figure 2). Additionally a graphical user interface for Microsoft Windows™ has been developed in Visual Basic.

### Input

There are three distinct input components: 1.) command line arguments to set program options or to override regular operation; 2.) the configuration file (an example is given in the file *config.txt* with explanation in the user manual, see ‘Supporting Information’), which provides settings for various program options and the names of additional files containing pertinent information; 3.) a primary input data file containing the MS data and additional data files, if needed, for a variety of information, such as original biomass (OBM) or the names for groups of biological replicates. The MS data consists of MDVs (Figure 1), all measured intensities or ion counts of the mass isotopomers of given analytes or fragments. If multiple chromatograms represent biological replicates they may be treated as individual datasets or averaged (after correction). Each supported compound is provided as a unique extension of the parent data type class. Each extension is named DataClass_XX.pm, where XX is a unique two character identifier for each supported compound class. The currently supported compounds are specified in Table 2. By following a modular approach, new compounds can be easily added using the existing compounds as a template with minor additions to the main program (to register a new identifier). The only additional information required is the elemental composition and structure of each analyte.

**Table 2:** **Overview of the analytes currently supported by *iMS2Flux *and the analytical platform on which they can be measured on**

**Analytes (with Acronym)**	**Analytic platform**	**Comments**	**Reference**
monomers from storage compounds:	GC-MS	compound specific derivatization, multiple analytes/multiple fragments	Allen et al. 2007, Junker et al. 2007
proteinogenic amino acids (AA) from proteins, glycerol (GY) and fatty acids (FA) derived from lipids, glucose (GL) from starch			
soluble metabolites (SM):	GC-MS	compound specific derivatization, multiple analytes/multiple fragments	Huege et al. 2007, 2010
sugars, amino and organic acids, et al.			
plant cell wall precursors (CW):	LC-MS	multiple analytes/single fragments	Alonso et al. 2010
sugars, sugar-phosphates and nucleotide-sugars			

The options available for processing the MS data depend on the actual data provided. A part of modularity in design includes the presentation of data, as such the main data format is text based, spreadsheet-compatible, tab-separated values (TSV). In this standard matrix the first column contains the analyte identifiers; the second column the mass of the respective mass isotopomers and the following columns the measured intensities for each chromatogram; the first row contains an optional title in the first element; the second row contains the identifiers for each chromatogram, an example is given in the file *Example_AA.txt* (see ‘Supporting Information’). This is a one dimensional data representation, meaning that each data file contains only a single type of data, such as raw MS measurements (typically expressed as ion counts or arbitrary units) or retention times. Therefore, to provide multiple types of data requires one TSV file per data type, otherwise there is no restriction on the type(s) of data that may be represented. In addition to the standard TSV format, third party/proprietary formats may be used through a custom import module. A third party module that is currently available extends the supported import formats to include the Waters Quanlynx™ report formats. For a detailed description of working through this format see the getting started guide; *MSto13C with QuanLynx and iMS2Flux.pdf* (see ‘Supporting Information’).

Due to the highly individualized nature of data extraction from MS chromatograms from different instruments, *iMS2Flux* does not extract MS data directly from chromatograms represented in proprietary formats. Instead one can use the extraction capabilities of third party software, e.g. TagFinder [30], MZmine 2 [31] or commercial solutions such as Waters Quanlynx™.

### Data checking

When implementing any form of automation it is crucial to thoroughly verify the quality of the original data. Thus before performing any data correction the program can perform several checks: first a simple check for missing values is performed; additional optional checks are: thresholds for minimum and maximum signal intensity (linear detector range), this is applied to all MS data including boundary data (see below and Figure 1), and deviations from the expected retention time window (specified as a number of standard deviations) of a respective analyte. Most data checks are performed on a per-fragment, per-chromatogram basis, with the exception of the retention time check. For statistical significance the mean and standard deviations of the retention times are calculated over all fragments and all chromatograms, which is valid for any set of chromatograms measured continuously in a given set of samples. A complete example illustrating the use of retention time analysis as a data check is provided in the appendix of the users’ manual (see ‘Supporting Information’). If any errors are found the program generates a list of the affected data and allows the user to review the data, going back to the chromatogram if necessary, manually edit or regenerate the data, and adjust the selected data check parameters if desired. Feedback is provided in TSV format, with any errors located in the position corresponding to the flagged data. In this way the error feedback can be overlaid as a mask on the measurement data facilitating user review of the flagged data.

Each chromatogram has to be composed of the same groups of MDVs. The first mass of these vectors is assumed to be the M+0 mass isotopomer (if not otherwise indicated in the configuration file), and is used to identify a fragment. Each fragment has a predetermined number of carbon atoms, and the last mass of a fragment is M+number of C-atoms (M+numC) (cf. Figure 1). For example, the molecular ion of alanine has a 3 carbon backbone, thus the last mass fragment would be M+3. It is also allowed to provide incomplete fragments. The default behavior of *iMS2Flux* is to expect that each fragment’s measurement value is provided in order. When an out of order mass is encountered *iMS2Flux* considers it to be the start of a possible new fragment. Thus, if a mass is missing in the middle of the MDV, *iMS2Flux* does not assume it is zero. Instead the fragment is treated as incomplete, and the remaining measurements belong to a non-existent fragment (and thus skipped). In such a case or whenever unknown data is encountered feedback is provided identifying where the problem was encountered. The program can also process boundary data around each mass fragment. If included, it can extract M-n and/or M+numC+n data points (Figure 1) which may be monitored for their relative value. To use either of these options all fragments must be consistent in the number of extra data points.

### Data correction

Each MDV is corrected separately and the resulting corrected intensities scaled to 100%, of the sum of all signals in the MDV. The corrections, if any, are applied in the order they are listed in the configuration file. If no corrections are specified, the uncorrected data are scaled and may be used as is.

**Figure 3:**
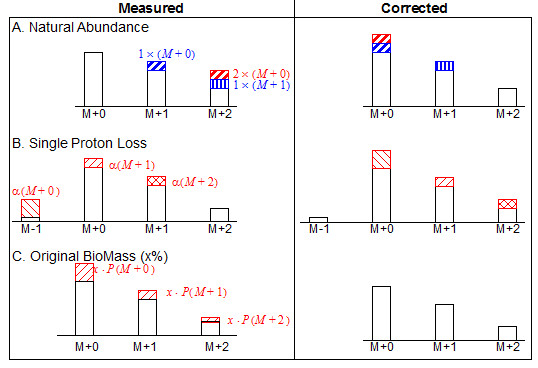
**Overview of MS data correction methods.** The comparison of MDV intensities is shown as they were measured (on the left) and as they are after applying the respective correction method (on the right). Also illustrated is the bias of the respective distortions: **A**) correcting for NOIs **B**) correcting for proton loss [the correction for proton gain follows the same principles] **C**) the influence the OBM.

Correcting for proton loss [36], see Figure 3b, or proton gain [37], is required when an individual hydrogen atom is lost or gained by the analyte during the MS process. Proton gain is characteristic for fatty acids when measured via GC-MS and adds mass to the McLafferty ion of fatty acid methyl esters [12,37,38]. For this correction it is assumed that the loss or gain affects a fixed percentage of the molecules, regardless of their labeling. This fixed percentage is called the scaling factor α. In the example illustrated in Figure 3b an analyte with a 2-carbon backbone is measured including the first preceding border mass, identified as mass M-1. The analyte suffers a single proton loss, causing a fixed percentage of each mass measurement to be artificially reduced, and for a non-negligible measurement of the M-1 mass, as described by the following set of equations:

$$\begin{array}{l} M_{-1}^{\text{meas}}=M_{-1}^{'}-\alpha M_{-1}^{'}+\alpha M_{0}^{'}\\ M_{0}^{\text{meas}}=M_{0}^{'}-\alpha M_{0}^{'}+\alpha M_{1}^{'}\\ M_{1}^{\text{meas}}=M_{1}^{'}-\alpha M_{1}^{'}+\alpha M_{2}^{'}\\ M_{2}^{\text{meas}}=M_{2}^{'}-\alpha M_{2}^{'} \end{array}$$

The non-linear set of equations is solved iteratively for the scaling factor, assuming the ideal case that the real boundary measurement is negligible. In the case where this assumption is not valid, it is possible for the scaling factor to be artificially inflated or for no stable value to be found. When no scaling factor is found the original data are left uncorrected. Similarly, correcting for proton gain requires the following border measurement, M+numC+1.

The main correction is for naturally occurring stable isotopes, or natural abundance (NA). Correcting for NOIs (Figure 3a), is performed for the specified set of elements that make up the measured analytes. For a given element, the NA of *N* atoms with *n* stable isotopes *I*_*i*_, each with probability *p(I*_*i*_*)* and occurring *f(I*_*i*_*)* times in the analyte is given by:

$$\text{Abundance}=N!\cdot \overset{n}{\underset{i=1}{\Pi }}\left(\frac{p{\left(I_{i}\right)}^{f\left(I_{i}\right)}}{f\left(I_{i}\right)!}\right)$$

Due to the diminishing probabilities of multiple heavy atoms occurring in one molecule by natural abundance, correction is performed only considering the labeling probabilities of the M+0, M+1 and M+2 isotopes of these elements [3-5,11]. A correction matrix is generated based on the number of atoms of each element considered and their natural isotope abundance [6,7]. The square size of the resulting matrix is related to the number of mass isotopomers, i.e. the size of the MDV, of the fragment being corrected.

For labeling experiments where the period of steady state metabolism during labeling is limited, the original biomass (OBM) makes up a significant percentage of the final biomass [13,14]. Under this condition it is necessary to remove the bias of the original unlabeled biomass from the labeling data. This correction for original biomass (Figure 3c) subtracts the OBM from the measured fragment [13,14] by using the natural abundances of the carbon isotopes (as used for NOI correction) to distribute the OBM over each measured mass isotopomer in a fragment. This correction requires an additional data file containing information of the percentage of OBM in each sample, an example is given in the file *OBM.txt* (see ‘Supporting Information’).

### Post correction checks

Once the MS measurement data has been corrected, it is possible to perform additional checks, such as for average labeling. Average labeling is the calculation of the average labeling due to the supplied label (e.g. ^13^C), and therefore must be performed after correction of the data. The average labeling is calculated on a per MDV basis as well as for an entire data set, and may be calculated for each chromatogram separately, or over each replica group. To be most useful the average carbon labeling must be performed on MS data from both labeled and unlabeled samples to identify possible contamination in individual MDVs, entire analytes or individual chromatograms. As with the other data checks the feedback is provided in matrix format (TSV) for manual analysis, allowing contaminated data to be excluded before further use. A complete example illustrating the use of average labeling is provided in the appendix of the user’s manual (see ‘Supporting Information’).

### Output

Output is generated at different stages of processing. If the raw data is to be output, MS measurements and retention times, this occurs immediately. Output may also be generated after the data checks and, as specified in the configuration file, at the completion of processing. All data types are generated in the standard TSV matrix, with one type of data per file. If feedback is generated during the (pre-correction) data check phase the program is terminated without performing correction. If the post correction data check is selected, output is always generated for both the average carbon labeling and the desired output as specified in the configuration file. Processed data is generated for each chromatogram with all boundary data removed. Even with no corrections selected, processed data for each fragment will still be normalized. In addition, if there is more than one replicate, basic statistics may be generated over each set of replicate data. For the calculation of statistics with replicate data the number of replicates in each group must be specified in the configuration file. Correspondingly, the experimental data for replicates must be given in consecutive order (column-wise) in the input MS data file, and in the same order as the number of replicates entered in the configuration file. In the case of no replicates, a default value (of five percent) is returned in place of the standard deviation to ensure compatibility (with MFA software that require an error value be entered). Optionally a file containing unique identifiers may be included for each experimental replicate set; otherwise generic file names are used for each replicate group.

In addition to the standard output format, *iMS2Flux* can generate data directly for use with third party MFA tools. Currently support is provided for the FTBL format for use with *13CFlux*[33] the CSV format of *OpenFLUX*[34] and the FML format of *13CFLUX2*[39]. For *13CFLUX* the MS measurement data can be generated for inclusion in either the ‘Mass Spectrometry’ or ‘Label Measurements’ sections of an FTBL file. This data can be generated into a set of individual files (one per set of replicates), or it can be directly included into one or more model files (of the specified format). To facilitate the use of analytes from different compound classes (such as amino acids and glucose) MS data can be appended to the existing MS data section of a given model file.

## Tool validation

*iMS2Flux* has gone through extensive validation of both the algorithms and the supported compound classes. In particular the central correction, for natural abundance, has been compared to both manually corrected measurements and those corrected by other correction tools. Manual correction compared both hand calculated correction and cut and paste with an Excel spreadsheet [38,40,41]. The algorithm was also compared with both the CORRECTOR [32] and to the MATLAB correction tool from Wahl et al. 2004 (afterwards called *MSCorr*) [11] correction tools. Minor variation was accounted for by different natural abundance fractions used in each method. Similarly the proton loss and gain were compared with the results in [38]. A complete set of uncorrected amino acid measurements from 24 experiments is provided with the software, together with the expected corrected values.

## Results and discussion

*iMS2Flux* has been designed to act as a high-throughput framework for MS data analysis, targeting MFA as its primary application, but is not inherently limited to MFA. The software is designed to be modular and flexible emphasizing a standard data exchange format. The standard data format allows for easy access through any spreadsheet application, and is supported with import and export modules to easily allow new tools to make use of the data. In Figure 2 an MFA workflow maximizing automation is illustrated, utilizing *iMS2Flux* to branch from data extraction to data analysis with ^13^C-Flux software (see ‘Supporting Information’). *iMS2Flux* defines not only the generic correction tool, but a fully standardized data format, and through it an automated workflow connecting third party extraction and analysis tools.

The performance of *iMS2Flux* was tested on a commercially available PC. The Perl interpreter was ActivePerl v.5.12.2 (from ActiveState). *iMS2Flux* is a non-threaded application and ran entirely on a single core. A set of GC-MS generated data comprising 128 chromatograms with a total of 65 fragments corresponding to 412 masses was processed in 119 seconds. To perform the benchmark *iMS2Flux* was set to check for missing data, detector threshold limits and poor peak values, extract an additional measurement (M+numC+1) from each fragment, perform natural abundance correction, generate the carbon labeling summary (post correction data check), generate average and standard deviations over replicates, and generate a complete set of output data (raw measurement, corrected measurement, average and standard deviation of corrected, and model data for each experimental set in *13CFlux* FTBL format for inclusion in the MASS_SPECTROMETRY section). The data was pre-screened to ensure that the MS data would pass all data checks to complete processing.

As illustrated in Table 1*iMS2Flux* offers a variety of options for data correction. Similar to *MSCorr*, it offers checks to ensure the MS data is within the upper and lower boundaries of the MS detector, whereas the tool *CORRECTOR*[32] assumes the process MS data is accurate. Depending on the tools used to extract the relevant MS data from chromatograms, e.g. [30,31,42] or manufacturer software, checks for data accuracy and quality can be performed during data extraction. *OpenFLUX*[34] is an MFA analysis tool that also provides a NOI correction tool (not directly integrated). Similar to *MSCorr*, the *OpenFLUX* correction is provided as a function in MATLAB (corrMatGen) which requires the user to enter the chemical formula and other specifics about each compound individually. *MSCorr,* corrMatGen*,* and *CORRECTOR* correct for NOIs, *iMS2Flux* allows additional correction methods: for original biomass as well as proton-loss or gain. Furthermore, *iMS2Flux* is capable of performing all the above mentioned corrections on large and heterogeneous data sets, comprising multiple analytes with multiple MDVs in multiple chromatograms. The addition of new analyte sum formulas in *iMS2Flux* is intuitive, since it only requires the total chemical formula of the new analyte, without separation of the metabolite derived part of an analyte from any derivatization reagent additions. Alternatively fully generalized analyte classes supporting multi-stage and multiple alternative derivatization are also possible. Finally the output of *iMS2Flux* is ready-to-use in MFA-dedicated software. The aforementioned *FiatFlux* is able to correct GC-MS data for natural abundance and original unlabeled biomass. The quality of the extracted MS data is checked in a similar way as in *MSCorr*, and faulty MS data can be removed manually from further calculations. Similar to *MSCorr* new compounds require a separation of the atoms of the analyte from the derivatizing agent. *FiatFlux* is focused on deriving flux ratios and absolute fluxes for microorganisms solely from 1-^13^C and/or U-^13^C glucose experiments combined with GC-MS analysis of amino acids [35].

Although *iMS2Flux* was designed to serve the needs of MFA, it can be used as a general tool to quantify stable isotope labeling in any kind of isotope tracer experiment, e.g. [32,40,43]. Furthermore, although carbon labeling with ^13^C is the method of choice in MFA, other elements such as nitrogen, hydrogen or oxygen are conceivable for tracer studies [44-46]. *iMS2Flux* can easily be adapted to any other element as isotope tracer. In order to allow the general application of *iMS2Flux* in MFA, independent of the MS platform the labeling data were acquired on, it was designed to process GC-MS, LC-MS or MS/MS data. Additionally, besides data from steady state labeling experiments, *iMS2Flux* can process dynamic labeling data as well. For the exploitation of the full potential of dynamic labeling experiments, such as short labeling time [47,48], it is necessary to be able to measure and evaluate MS data not only derived from metabolic end products (storage compounds) but from metabolic intermediates, which can have a very fast turnover [49,50]. This would increase the resolution of a metabolic network and can resolve precursor-product relationships which are difficult or impossible to resolve with data derived from end product labeling [11]. To give *iMS2Flux* this capability, data of the elemental composition of polar soluble intermediates of primary metabolism, as previously published [32,41], were included. This list of supported analytes can be extended as needed, in case new metabolites are of interest or a different derivatization strategy is applied.

In the context of measuring complex biological matrices of soluble metabolic intermediates, similar to metabolic profiling measurements, it seems appropriate to use specialized software. Since there are multiple software solutions available, especially dedicated to the alignment of multiple MS chromatograms and extracting the relevant MS data, e.g. [30,31,42] or manufacturer software, our efforts focused on finding a general input format that supports the respective data outputs, the TSV format described above. *iMS2Flux* was implemented in PERL which is freely available and runs on all major computing platforms. Furthermore, MS data are usually provided in tabular form, which is either already in the TSV format, or is easily exported to TSV, thus a text manipulation language was the obvious choice. PERL supports multiple programming paradigms and no compilers are required, as it is a dynamic language a respective script just needs to be edited and can be run directly. To further promote the use of *iMS2Flux*, the code is provided in full and since the program is not compiled the source is immediately available to be reviewed and extended for individual needs. To support flexibility the different data formats, optional data checks, data correction and output formats are contained in individual modules.

## Conclusions

With *iMS2Flux* we have developed an MS data processing tool for isotope labeling experiments with special focus on increasing throughput at multiple stages of the data analysis pipeline. Thus from the computational side MFA technology is now ready to be applied on a large scale, as is already common in the other –omics methods. By using *iMS2Flux* in our daily work we found that by liberating the researcher from the most laborious tasks of MS data processing, *iMS2Flux* removes the limitations on the number of samples that can be processed per tracer experiment, including the number of treatments or genotypes studied, the replication of each experiment, the number of substrate combinations used, and/or the number of time points analyzed. This increases the accuracy and coverage of MS data; in turn this has the potential to improve the accuracy (including overdetermination) and scope of MFA and flux profiling and its integration into multiomic systems biology.

## Availability and requirements

**Project name:***iMS2Flux*

**Project home page:**http://sourceforge.net/projects/ims2flux

**Operating system(s):** Platform independent

**Programming language:** PERL

**Other requirements:** PERL v.5 or higher

**License:** This work is licensed under the Creative Commons Attribution-NonCommercial 3.0 Unported License. To view a copy of this license, visit http://creativecommons.org/licenses/by-nc/3.0/ or send a letter to Creative Commons, 444 Castro Street, Suite 900, Mountain View, California, 94041, USA.

**Any restrictions to use by non-academics:** license needed.

## Supporting information

The *iMS2Flux* software and all auxiliary files and instructions can be downloaded from the SourceForge project website: http://sourceforge.net/projects/iMS2Flux

The main download is a zip file, iMS2Flux.zip. When unzipped it will create a folder iMS2Flux containing the following:

• *readme1st.txt* – a brief introduction and a complete listing of the directory structure.

• *iMS2Flux.pl* - the main program,

• *iMS2Flux-Manual.pdf* – the user's manual,

• *Example_AA* – a folder containing all of the example files, including a copy of the expected results when running the example using the instructions in the relevant getting started guide.

• *FluxY_Lib* – a folder containing the program libraries common to several projects in the larger FluxY toolset,

• *Getting_Started* – a folder containing instructions on getting started installing and using the software on different platforms, and

• *Math* – a folder containing the CPAN library used by *iMS2Flux*.

## Competing interests

The authors declare that they have no competing interests.

## Authors’ contributions

CHP, JH, YSH and BJ conceived the software. CHP implemented the software. CHP and MF implemented the graphical user interface. CHP and JH drafted the manuscript. MF and CK critically evaluated the software. CK, YSH and BJ revised the manuscript. All authors read and approved the final manuscript.
